# Free breakfasts in schools: design and conduct of a cluster randomised controlled trial of the Primary School Free Breakfast Initiative in Wales [ISRCTN18336527]

**DOI:** 10.1186/1471-2458-7-258

**Published:** 2007-09-21

**Authors:** Laurence Moore, Graham F Moore, Katy Tapper, Rebecca Lynch, Carol Desousa, Janine Hale, Chris Roberts, Simon Murphy

**Affiliations:** 1 Cardiff Institute of Society, Health and Ethics, Cardiff University, 53 Park Place, Cardiff, UK; 2 School of Psychology, Swansea University, Singleton Park, Swansea, UK; 3 Public Health Strategy Division, Public Health and Health Professions Department, Welsh Assembly Government, UK

## Abstract

**Background:**

School-based breakfast provision is increasingly being seen as a means of improving educational performance and dietary behaviour amongst children. Furthermore, recognition is growing that breakfast provision offers potential as a means of addressing social inequalities in these outcomes. At present however, the evidence base on the effectiveness of breakfast provision in bringing about these improvements is limited.

**Methods/Design:**

This paper describes the research design of a large scale evaluation of the effectiveness of the Welsh Assembly Government's Primary School Free Breakfast Initiative. A cluster randomised trial, with school as the unit of randomisation was used for the outcome evaluation, with a nested qualitative process evaluation. Quantitative outcome measures included dietary habits, attitudes, cognitive function, classroom behaviour, and school attendance. The study recruited 111 primary schools in Wales, of which 56 were randomly assigned to control condition and 55 to intervention. Participants were Year 5 and 6 students (aged 9–11 years) in these schools. Data were collected for all 111 schools at each of three time points: baseline, 4 month and 12 month follow-up. This was achieved through a repeated cross-sectional survey of approximately 4350 students on each of these occasions. Of those students in Year 5 at baseline, 1975 provided data at one or both of the follow-ups, forming a nested cohort. The evaluation also included a nested process evaluation, using questionnaires, semi-structured interviews and case studies with students, school staff, and local authority scheme coordinators as key informants.

**Discussion:**

An overview of the methods used for the evaluation is presented, providing an example of the feasibility of conducting robust evaluations of policy initiatives using a randomised trial design with nested process evaluation. Details are provided of response rates and the flow of participants. Reflection is offered on methodological issues encountered at various stages through the course of the study, focusing upon issues associated with conducting a randomised trial of a government policy initiative, and with conducting research in school settings.

**Trial registration:**

Current Controlled Trials ISRCTN18336527

## Background

Many school children do not eat breakfast everyday: in a survey of year 6 children (aged 10–11) conducted in the United Kingdom, 5% of children reported having not had anything for breakfast that day, 3% had only consumed a drink and approximately a further 10% reported eating crisps or chocolate for breakfast [1]. Not eating (or 'skipping') breakfast has been associated with a wealth of deleterious health outcomes [2-4] poorer overall nutritional adequacy [5,6] and detrimental effects upon memory and concentration [5-9].

A recent study amongst schoolchildren in Wales focusing upon associations between physical activity, dietary behaviours and obesity indicated that breakfast was the most commonly missed meal amongst all children, with obese children skipping breakfast on average twice a week, double the frequency of normal weight children [10]. Furthermore, studies conducted in the United Kingdom demonstrate a significant social gradient in breakfast eating habits, with children from more deprived backgrounds more likely to skip breakfast than their wealthier counterparts [11-13]. In addition, consumption of poorer quality breakfasts has been found to be associated with higher levels of deprivation, as have unhealthy attitudes towards breakfast [12]. Therefore, promotion of habitual and healthful breakfast eating behaviours may promote population health and academic performance and reduce health and educational inequalities.

In recent years, many efforts to facilitate change in dietary behaviours have been directed towards schoolchildren due to the capacity of such approaches to reach large numbers of children simultaneously [14,15]. Habitual behaviours developed in childhood may track into adulthood, with potential consequences for health later in life [16], and repeated exposure to healthier foods at an early age has been shown to increase the intrinsic rewards associated with their consumption [17-20].

Appropriate manipulation of the school environment may offer an efficient and effective long-term means of improving the health of the population. Furthermore, if such intervention brings about sufficient dietary improvement to impact upon cognitive functioning and behaviour [21], this may translate into improved school performance and educational achievement.

Recognition of these potential benefits has led to a number of government funded school breakfast initiatives (e.g., see Shemilt et al [22]). These were first employed in North America in 1966 and aimed to improve the nutritional status of children in deprived areas [23]. Since then, the number of schools participating in such programmes has risen dramatically, so that by 1997 approximately six million children in the US were attending a school breakfast club each day [24]. In the UK, the introduction of breakfast programmes has occurred more recently, with funding from sources such as the Education Action Zone initiative, New Opportunities Funding and Sure Start out of School Funding. The Department of Health also introduced a pilot initiative in 1999. However, the objectives of these different breakfast clubs have varied considerably; whilst some have focused on the provision of a healthy breakfast, others have placed more emphasis on childcare, education, or informal interaction between children and school staff [22]. Indeed, a "breakfast club" may not necessarily provide breakfast.

Nevertheless, there is evidence to suggest that school breakfast programmes can help improve nutrition and may also be associated with improvements in attendance, academic performance and behaviour (e.g., [7,25-28]). However, findings have been inconsistent [23] and the research has had limitations, with most of the studies unable to incorporate appropriate control groups or suffering substantial contamination between trial arms. Thus although there is good reason to believe that breakfast programmes can have a wide range of beneficial outcomes, this has yet to be convincingly demonstrated.

### The Primary School Free Breakfast Initiative

The Welsh Assembly Government's Primary School Free Breakfast Initiative arose from a Labour Party manifesto commitment to make free healthy breakfasts available to all maintained primary schools in Wales. This paper reports upon the design of a comprehensive national evaluation of this initiative, using a cluster randomised controlled trial design with a nested process evaluation, and discusses issues in the implementation of the evaluation design.

## Methods/Design

### Study design

The evaluation study comprised of two key components. In order to evaluate the overall effectiveness of the intervention, a randomised controlled design was employed. Randomised controlled trials (RCTs) are generally considered the most reliable means of assessing intervention efficacy (e.g., see Campbell et al [29]). Since public health interventions, such as the school breakfast initiative, tend to act at multiple levels and through multiple channels [30] implementation of a traditional RCT is difficult. It is likely that these methodological difficulties have contributed to the absence of good evidence for the efficacy of school breakfast programmes and other public health interventions. However, recent developments in research methodology, including the use of cluster RCTs and mixed methodologies have the potential to provide unbiased estimates of the effectiveness of such interventions as well as identifying other factors that are more variable such as intervention delivery, context and support [31].

**Figure 1:**
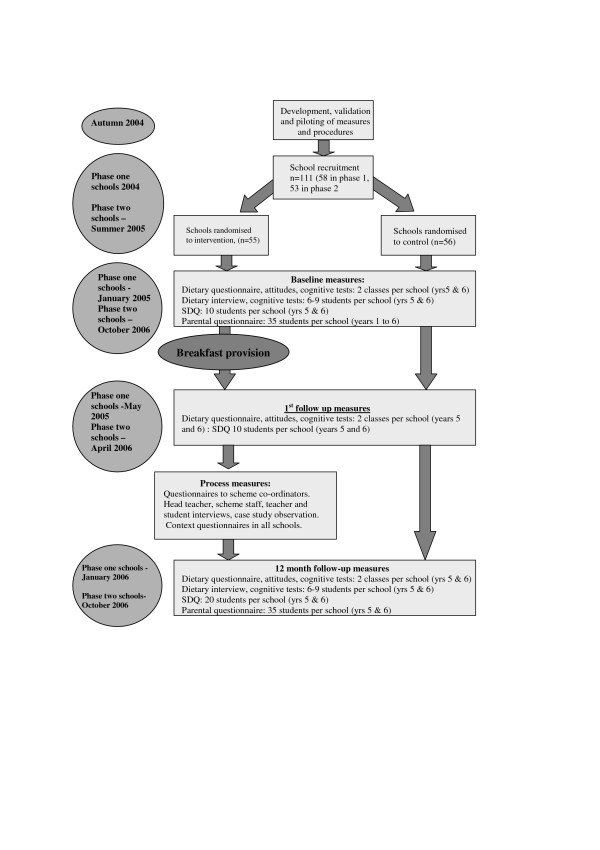
Timetable for evaluation of the Welsh Assembly Government's Free School Breakfast Initiative.

As the intervention was implemented at the school-level, randomisation of individual students to control or intervention groups was not practicable. As such the evaluation adopted a cluster randomised design with school as the unit of randomisation. A summary of this study design is presented in Figure 1. In line with the aforementioned development of mixed-methodology approaches to evaluation, a nested qualitative process evaluation was included to address issues concerning the context and implementation of the initiative. In this way, the evaluation not only addressed the question, 'Does it work?', but also considered 'What works?', 'For whom?' and 'Under what circumstances?' [32].

### The Primary School Free Breakfast Intervention

The intervention provided a school-based breakfast before the commencement of classes, without any cost being borne by parents. The aim of the intervention was not only to encourage breakfast consumption per se, but to improve the nutritional quality of children's breakfasts. Hence a particular focus was placed upon limiting available food choices to breakfast items considered to be healthful. Provision was therefore offered from four food types: non-sugar coated cereals, bread, milk products and fruits, in addition to drinks. Schools were provided with guidelines regarding how the scheme should be run, in terms of staff to student ratios and the food to be provided, but were given a reasonable degree of autonomy in the operation of the scheme.

The scheme was rolled out in two distinct phases by the Welsh Assembly Government, with initial priority given to schools in more deprived areas of Wales. As such, schools classified as 'Communities First' schools were eligible to receive the intervention initially before the scheme was made available to all schools the following year. At the time of writing, information about the scheme can be found on the Welsh Assembly Government's website [33].

### Recruitment and randomisation of schools

#### Phase one – Communities First schools

In autumn 2004, head teachers of infant, junior and primary schools located in 'Communities First' (i.e. deprived) areas in nine local education authorities (LEAs) in north, south and west Wales were invited to participate in the evaluation. Schools were offered £250 to compensate for additional teacher time and disruption to school activities. One hundred and fifty-two schools were approached and 58 schools agreed to participate. Reasons for non-participation were generally related to the running of the breakfast scheme itself (worries about implementing the new initiative and bringing forward the start date) rather than any concerns over data collection burden. Those that agreed to participate were randomised to the intervention or control condition using strata defined by LEA, school size, free school meal entitlement and Welsh language medium.

#### Phase two – non-Communities First schools

Head teachers of infant, junior and primary schools located in 'Non-Communities First' (i.e. more affluent) areas, in the same nine LEAs were invited to participate in the evaluation. As before, schools were offered £250 to compensate for additional teacher time and disruption to school activities. This time, 456 schools were approached and 53 schools agreed to participate. Again, reasons for non-participation were generally related to fears about relinquishing control over the start date of the scheme, although a number of differences in reasons for non-participation unique to this phase of the study were identified (see response biases section of Discussion). Those who agreed to participate were randomised to intervention or control following the same methods of stratification as described above.

For both phases, schools in the intervention group were asked to set up a breakfast scheme, following the guidance issued by the Welsh Assembly Government, after baseline measures had been collected. Schools in the control condition were asked to refrain from setting up a breakfast scheme until after the 12 month measures had been collected.

In each of these 111 schools, following the timetables set out in Figure 1, one class of Year 5 children (9–10 years) and one class of Year 6 children (10–11 years) were randomly selected to complete class based measures. From this sample, 5 children from each year (i.e. 10 in total) were randomly selected to be assessed by teachers (see below) and 6–9 of these children were randomly selected to participate in individual testing. In addition, for each school 35 children were randomly selected from the full age range, and a questionnaire was sent to their parents. For primary schools, 5 children were sampled from each year group (i.e., reception to year 6) and for junior schools, 8 or 9 students were selected from each year group (i.e., years 3 to 6).

### Outcome measures

The quantitative measures employed in the evaluation aim to provide an accurate assessment of the impact of the scheme on children's dietary habits, cognitive performance, attitudes and classroom behaviour. As previously discussed, since the intervention is implemented at the school-level, randomisation has to take place at this level. As a consequence, relatively large numbers of participants are needed in order to detect any intervention effect. For such a trial to be feasible it is therefore important that the outcome measures employed are relatively quick, cost-effective and easy to implement. In an attempt to address the inevitable tension between measurement precision and sample size/response bias (i.e., brief measures, whilst cost effective and perhaps encouraging higher response rates, are likely to suffer from a higher degree of measurement error) our evaluation incorporated two levels of assessment. For dietary measurement, the study first used a less sensitive dietary recall questionnaire administered simultaneously to the whole class. Secondly, a subsample of children also participated in a validated, and much more time consuming, one-to-one dietary interview procedure.

The following standardised, previously validated measures were used:

#### Cognitive tests

Classroom administered cognitive tests were used to assess episodic memory, working memory, sustained attention and psychomotor speed [34-36]. Individually administered, computerised cognitive tests were used to assess sustained attention, selective attention, simple reaction time and choice reaction time. The most consistent effects of breakfast upon cognition, in experimental conditions, have previously been observed for episodic memory [35,37,38] which was therefore selected as the primary outcome in terms of cognitive function.

#### Strengths and Difficulties Questionnaire

The Strengths and Difficulties Questionnaire [39] was completed by teachers to assess children's classroom behaviour. This was a relatively brief, but well-established measure that assessed five dimensions of behaviour: hyperactivity, emotional symptoms, conduct problems, peer problems and pro-social behaviour. Hyperactivity in particular, is likely to be influenced by breakfast due to its relation to on-task behaviour [40]. Therefore this sub-scale will be analysed as a secondary outcome, with the global total difficulties scale analysed as a tertiary outcome.

#### Dietary recall interview

Individually administered dietary recall interviews were used to provide a more accurate estimate of the impact of the initiative on children's diets. These interviews were conducted using a standardised protocol based on that employed by Lytle et al. [41].

Due to a shortage of previously validated measures the following measures were developed and validated for use in the present study [42,43]:

#### Dietary recall questionnaire

Children were asked to list all foods and drinks consumed at chronologically ordered time points throughout the day (e.g., at home before school, on the way to school, at school before class started). Details of breakfast on the day of reporting (i.e., any foods consumed before the start of classes) were collected first, followed by details of the previous day's intake [42]. Primary measures from this questionnaire are the number of healthy food items (i.e., fruit, bread, cereal and milk products) and number of unhealthy food items consumed at breakfast (i.e., sweet items and crisps), and the number of days on which breakfast was consumed in the last two days (i.e., 0, 1 or 2).

#### Attitudes towards eating breakfast

Attitudes were assessed using a questionnaire containing thirteen statements referring to a variety of domains, such as concentration and behaviour, energy, and the general importance placed on breakfast. Children were asked to indicate the extent to which they agreed or disagreed with each statement by placing a tick in one of 5 boxes (agree a lot/agree a bit/don't agree or disagree/disagree a bit/disagree a lot) [43].

#### Parental questionnaire

The parental questionnaire contained 10 questions designed to assess children's breakfast eating habits. Five of these asked parents how many times on school days their child usually engaged in a particular behaviour (ate breakfast at home, took something from home for breakfast to eat on the way to school or at school before the start of class, took money to buy breakfast on the way to school, ate a breakfast provided by the school, missed breakfast). These were answered by placing a tick in one of 7 boxes (number of days ranging from 0 to 5 or 'Don't know'). Four questions asked parents how many times at the weekend their child usually engaged in a particular behaviour (ate breakfast at home, took something from home for breakfast to eat elsewhere, took money to buy something for breakfast, missed breakfast). These were answered by placing a tick in one of 4 boxes (number of days ranging from 0 to 2 or 'Don't know'). An additional question asked parents to rate the frequency with which they thought their child ate a healthy breakfast. This was answered by placing a tick in one of 5 boxes ranging from 'My child always eats a healthy breakfast' to 'My child rarely eats a healthy breakfast'.

### Sample size calculations

Since there are a range of outcome measures, sample size requirements were calculated using effect sizes. Sample size calculations assume an intra-cluster correlation of 0.02, 80% power, and a two-tailed alpha of 0.05. With 111 schools in the trial, for pupil outcomes from the self-complete questionnaire, assuming an average of 50 responses per school, there will be power to detect an effect size of 0.11. For parent reports of breakfasting behaviour, assuming 20 responses per school, there will be power to detect an effect size of 0.15. For pupil outcomes from the dietary recall interviews, there will be power to detect an effect size of 0.2.

### Data collection

Parents were informed of the research in advance by means of a letter and information sheet either posted to them or sent home with children, and were asked to complete a return slip and/or contact the school if they did not wish their child to participate in the study. At each data collection, children were also informed that they were under no obligation to participate. The study received ethical approval from the Cardiff University Social Science Ethics Committee.

Cognitive tests, the attitudes questionnaire and the dietary recall questionnaire were completed between 9 am and 12 pm as supervised classroom exercises with a maximum class size of 40 children. For the attitudes questionnaire, to minimise conferring and ensure that children worked at the same pace and did not distract one another, the researcher read out the statements one by one and children marked their response for each statement after it was read out. For the dietary recall measure, the researcher read out the instructions and asked children to complete the questionnaire independently from one another. Children were asked to put their hands up when they had finished or if they needed help with spelling, or further clarification of questions. Three members of the research team were present to assist children.

As described above, at baseline and 12-month follow-up only, 3–5 children from each year group (e.g. year 5 and 6) were selected to complete individual tests between 12:30 and 3:30 pm (although timetabling restrictions occasionally led to these being conducted as early as 11:00 am). These were conducted on a one-to-one basis with one researcher guiding each child through the computerised cognitive tests and the dietary recall interview.

Schools were divided between key members of the research team, and each key researcher was responsible for all liaison with their assigned schools, and held principal responsibility for data collections. Two trained temporary research assistants were also brought to each school at baseline and 12-month follow-up, to provide general assistance with group testing and to conduct individual testing in the afternoon. For the first follow up, only one assistant was required as only the morning's group testing was carried out. All temporary research assistants were fully trained and monitored by the key researchers in order to maximise standardisation across the trial sites and data collection sweeps.

### Statistical analyses

There was no one primary outcome, but a pre-specified analysis plan was agreed in which the following were identified as primary outcomes: the proportion of students consuming two breakfasts over two days; episodic memory; number of healthy food items consumed at breakfast and number of unhealthy food items consumed at breakfast according to the dietary recall questionnaire. Secondary outcomes were identified as attitudes towards eating breakfast; rest of day healthy food items; rest of day unhealthy food items; scores on the hyperactivity/inattention scale of the strength and difficulties questionnaire, and parental reports of frequency of eating breakfast at home and at school. A number of other outcome measures were *a priori *defined as tertiary outcomes, including cognitive measures other than episodic memory, the total difficulties score on the strengths and difficulties questionnaire, parental reports of morning routines and child care arrangements before school and variables collected from the individually administered in-depth dietary recall and cognitive measure interviews.

For each outcome variable, the primary analysis is a school-level weighted regression analysis [44] adjusting for baseline score and the four stratification variables. These primary analyses will be conducted on an intention-to-treat basis, in which each school is coded according to the treatment condition to which it has been randomised. A secondary analysis conducted for all variables will be a per protocol analysis, in which schools are coded according to whether or not a free breakfast scheme was set up prior to outcome measurement.

Sub-group analyses are planned for study phase, socio-economic status and groups defined by consumption of breakfast, healthy and unhealthy food items at baseline. Socio-economic status will initially be defined in terms of a school level variable indicating the proportion of students entitled to free school meals, but through further data linkage it is planned that student level socio-economic measures based on postcode of residence will be available. If this additional data linkage is undertaken, it will also allow the performance of students in Statutory Assessment Tests and public examinations to be included as additional (secondary) outcome variables, although this is not included in the current protocol as it is dependent on ethical approval and funding. Other sub-group analyses may be conducted according to variables identified in the process evaluation, which may include measures on the implementation of the scheme or relating to the school and community context.

### Process evaluation

Whilst recognising the need to adopt appropriate research designs and to draw on theoretically informed outcome measures to evaluate complex interventions, it is of equal importance to understand processes [45]. The evaluation therefore incorporated a substantial process element that examined how the initiative had been implemented. This will facilitate the interpretation of outcome effects and add to the understanding of how a major policy initiative is undertaken.

An initial preliminary process evaluation was also completed with schools who began provision of free breakfasts during the first wave roll out in September 2004. This consisted of telephone interviews with the Welsh Assembly Government breakfast team, LEA co-ordinators and questionnaires with school based co-ordinators. In addition, a number of case study schools were selected for an in-depth observational and interview study with teachers, caterers and students. This preliminary study allowed process measures and an appropriate design to be developed and piloted before the main process evaluation was undertaken. Results also formed the basis of a report to the Welsh Assembly Government on the initial implementation with recommendations for policy and practice [46].

The main process evaluation consisted of telephone interviews with LEA co-ordinators, a postal questionnaire for school co-ordinators of the scheme and interviews and observation in case study schools. Following lessons learnt from the preliminary study, the process evaluation drew on the framework proposed by Steckler and Linnan [47] to examine the following areas:

1. Context. Existing dietary health promotion activity to determine the relationship between school climate, implementation and outcomes [48].

2. Fidelity and dose. Details of initiative content and a comparison of daily record keeping for the Assembly Government and for guidance documentation.

3. Integration. Details of existing, and any changes to, school policies and the level of integration with curriculum and extra-curricular activities.

4. Recruitment, participation and reach. Approaches to promotion and recruitment, any use of targeted recruitment, level of, and explanation for, participation and non participation.

5. Implementation and sustainability. Barriers and facilitators to implementation, level and type of staff involvement, level and type of parental involvement, in and out of school time involved, direct and indirect financial costs, benefits and costs to school, staff, students and parents.

The case study schools consisted of eight participating schools from each phase of the evaluation that were purposefully selected to explore these issues in greater depth. Schools were chosen so that differences in size of school, local education authority, setting (e.g. urban or rural) and percentage of free school meal entitlement were reflected. School selection was determined by information gathered from the teachers, uptake records and school characteristics. In this way a range of approaches to, and experiences of, implementation were examined. In these schools, semi-structured interviews were conducted with head teachers, teachers, breakfast scheme staff and students attending the scheme. The interviews asked for accounts of any changes to the school, dietary behaviour, school behaviour, attitudes and norms as well as views on, and experiences of, the initiative and its implementation. Breakfast schemes were observed in each of the case study schools with observational records taken of the delivery of the intervention.

Finally a questionnaire was sent to head teachers in all participating schools asking about policies, initiatives and contextual issues that had occurred over the course of collecting the quantitative data. In this way, events that may have affected the outcome data could also be taken account of.

### Participation rates and school attrition

Of the schools where baseline data were collected, no schools withdrew from the study prior to final data collection. There were however five schools randomised to the control group who nevertheless set up a free breakfast scheme prior to the 12-month follow up, while ten schools randomised to the intervention group had not set up the scheme within the follow-up period. Thus the per protocol analyses have 61 schools coded as control schools and 50 as intervention.

#### Participation of students

Response rates for students participating in classroom data collections are presented in Table 1. Whilst there was some minor variation between measures in terms of responses, with for example some children arriving late in class having missed one measure or having to leave before the final measure had been completed, these differences were negligible. Response rates for cognitive testing ranged from 85.5 to 87.9%, for the attitudes measure from 86.3 to 88.3% and for the dietary recall questionnaire from 86.2 to 88.4%. For ease of reading, detailed response data relating to only the attitudes towards breakfast questionnaire are presented, with the choice of measure entirely arbitrary.

**Table 1:** Response rates and reasons for non-participation amongst students within control and intervention schools, at baseline, 4-month and 12-month follow up

	Intervention			Control		
	
	DC1	DC2	DC3	DC1	DC2	DC3
Sampled	2484	2533	2569	2442	2437	2493
Parent refusal	12	0	2	6	0	0
Left school	9	34	60	11	20	41
Eligible	2463	2499	2507	2425	2417	2452
Data collected	2205	2201	2272	2145	2157	2200
Data missing/invalid	18	13	5	14	8	31
Excluded due to special educational needs	37	19	11	23	16	7
Child refusal	3	0	2	3	6	7
Absent	200	266	217	228	230	205
Class unavailable on day of testing	0	0	0	12	0	0

For both control and intervention schools, and across all 3 data sweeps, data were collected from more than 85% of those eligible to take part, with the majority of non-participation due to the child's absence from the school on the day of testing. Only very small numbers of children were excluded either by parents prior to data collections or by their decision not to give consent on the day of testing. The variation between the numbers of children sampled at each sweep reflects the clustered nature of the sampling strategy, with repeated cross sections sampled from within each school, varying marginally from one time point to the next due to changing class compositions.

**Figure 2:**
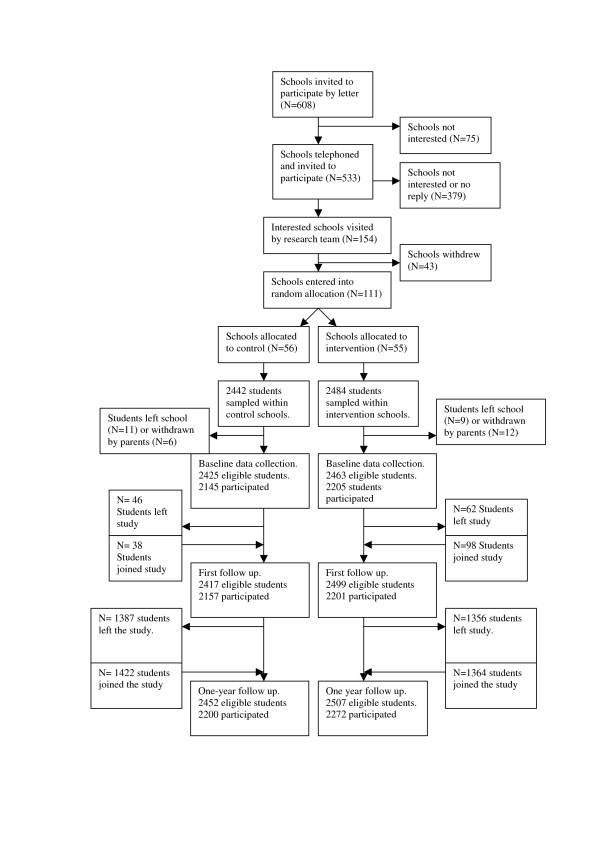
Flow of participants through the study.

The flow of participants through the study is presented in Figure 2. The inflow and outflow of participants between the first and 12 month follow ups reflects the fact that the follow-up of each school at 12 months is in effect a repeated cross-section design from the perspective of students. Thus, approximately half of the cohort recruited for the baseline and first follow-up measures are replaced at 12 months by an incoming group of year 5 students. These repeated cross-sections will be used for school-level analyses which are the primary analyses for the trial. Additional research questions may be addressed through analysis of the nested cohort of participants eligible to participate at all 3 time points, of whom there were 1975 who provided data at baseline and one or both of the follow-ups.

## Discussion

### Key issues in conducting the evaluation

A number of key issues arising from this, and similar, evaluations are likely to be of interest to those attempting to undertake similar work. Developing and maintaining an effective working relationship with schools is crucial to a project involving such long-term commitment and intrusion into their operation. Related to this, efforts to minimise attrition rates are crucial. Further potential difficulties arising from attempting to align a research timetable with an externally defined policy timetable include response biases and contamination. A number of ethical and operational issues surround gaining consent from students and parents and the undertaking of data collections in primary schools settings.

### Working with schools to conduct research

It is important to remember that schools' involvement in a large-scale trial such as this requires significant voluntary co-operation and commitment from a group of professionals with many conflicting demands and priorities. The trial of the Primary School Free Breakfast Initiative required both a considerable relinquishment by schools of control over when to set up the initiative, and a commitment to participate for a period of over one year. Given these concerns, the importance of maintaining a positive working relationship with schools is paramount. Principles for maintaining such relationships are laid out by Peterson et al [49], including maintaining regular contact and keeping schools up to date with relevant information, minimising the burden of work for schools, responding to the needs of the school and expressing gratitude for participation (p.154).

These principles were followed throughout this trial from recruitment onwards. Once the schools expressed an interest in taking part, a research team member visited the school and discussed any concerns that they may have, before the head teacher either agreed to set up the scheme or withdrew interest. This meeting was intended to ensure that schools were fully informed and aware of their commitments as participants, as well as providing an opportunity to address any concerns. Some considerable work on the part of the schools was required, in terms of for example completing questionnaires and twice labelling 35 parental questionnaires with addresses (as we could not ask for these addresses ourselves due to data protection restrictions). Hence, it was important to make clear from the outset exactly what was expected, and that the payment for taking part was our way of expressing gratitude for this dedication.

This visit, from the research team member who would be leading data collections in that school, helped to humanise the project, providing the school with a contact person for any concerns regarding the study. Where possible, this contact remained constant throughout, though in many cases this was not possible due to staff turnover. For schools wishing to participate, contact details were provided for an LEA representative to speak to about operational issues surrounding the scheme. Over the course of the following year, the research team maintained regular contact with schools, sending newsletters to keep schools up to date with the trial's progress and thanking them for their involvement.

### Attrition

Whilst issues surrounding attrition may arise from the relationship developed between schools and the research team, in the context of evaluating an initiative such as this one, where schools are asked to relinquish control of start dates for an otherwise available initiative, external pressures and difficulties may arise for schools at a later date which influence their ability to fulfil these obligations. These need to be dealt with sensitively on a one-to-one basis.

Whilst no schools refused to allow researchers into the school to collect all three rounds of data, a number of intervention schools experienced significant difficulties in setting up the scheme and ten were unable to set-up prior to follow-up data collections. All LEAs were informed which schools were in the control condition and were advised that these schools had agreed not to set up until after 12 month follow up. However, a number of control schools experienced external pressures from parents and other sources to set up the scheme earlier than agreed, which they felt unable to resist. In these instances, the research team attempted to either negotiate with schools to wait until the agreed date, or if such an agreement could not be reached, data collections were brought forward to a slightly earlier date to allow set up to take place earlier. In five cases, schools set up the scheme before these measures could be put in place. Data were still collected from all schools thus allowing intention to treat and per protocol analyses to be undertaken.

### Response biases

Response biases, whilst likely to be minimal at the individual level due to the high rates of participation amongst children, may occur at the school level. Schools choosing not to set up the breakfast scheme may differ systematically from those recruited to the trial. Furthermore, as the intervention was to become available to all schools regardless of their participation within the trial, those wishing to set up the scheme but choosing not to participate in the evaluation may also differ from evaluation study participants. Data were routinely collected on school characteristics such as size, deprivation levels (as indicated by the percentage of children receiving Free School Meals) and language of teaching. These were used as criteria for stratified randomisation and will be used to establish representativeness.

Factors relating to the timing of intervention implementation may have impacted upon responses, and there were a number of contrasts between the first and second phases of the evaluation in terms of these issues. As discussed, the manifesto commitment underpinning the initiative's rollout ensured that it would become available to all schools in due course. However, in the first phase (Communities First Schools), participation in the trial provided the prospect of starting the intervention earlier than general roll-out. By contrast, in the second phase (Non-Communities First schools), the initiative began to be rolled out to non-participating schools as the evaluation began, and hence schools who strongly wished to set up the scheme in their school were perhaps likely not to take part in case they were assigned to the control condition and were required to wait a further year. Furthermore during the second phase of the study, some head teachers appeared anxious to set up the initiative as soon as possible, with many nearby schools having already established the scheme. Breakfast provision was seen by some as a factor which influenced parental choice of school, hence impacting upon school numbers. Conversely, some heads, having observed the experiences of others, had made the decision not to set up the scheme in their school. Reasons for non-participation have been collected wherever possible, and these will be used to explore such issues in greater depth.

### Contamination

Issues surrounding the provision of information and awareness of the scheme amongst control schools cannot be overcome in the context of such a large-scale evaluation of a high profile government initiative. However, to minimise contamination, schools in the control arm of the intervention were asked not to partake in any form of marketing for the scheme until after final follow up data were collected. In a number of instances, the Welsh Assembly Government distributed promotional information to all Welsh primary schools regarding the initiative. Efforts were made to ensure that this information was not distributed to schools within the control arm.

### Consent

Consent for participation in the trial was sought at three levels. Firstly, consent for the schools participation was sought in the form of a signed agreement between headteachers and the research team. Secondly, consent was sought from parents. Rather than written, opt-in consent which is the established practice in clinical trials, a standardised information sheet was sent to all parents accompanied by a letter requesting that the parent contact the research team or school prior to data collection, should they wish to exclude their child from the study. Passive, opt-out consent is favoured over opt-in consent in order to ensure high response rates and minimise response biases, and has been described as an ethical and appropriate way of informing parents of 'low-risk' research [50].

The third and final level of consent was sought from children themselves on the day of data collection. Prior to any data being collected, procedures were described to children and children were all asked whether they were happy to take part, and it was made explicitly clear that they were under no obligation to if they did not wish to do so. This combining of parental and child consent has been used effectively in similar school-based cluster-randomised controlled trials [50].

### Data collection

A number of important issues arise in collecting data in a school setting. As with any form of work with children, child protection issues are paramount, and as such, all data collections were conducted by lead researchers who had been cleared by the Criminal Records Bureau, accompanied by one or two assistants. This small team of researchers would remain together throughout the day, with no single person ever left alone with individual children or groups of children.

Secondly, issues of confidentiality must be dealt with effectively, which is inherently difficult in the context of a trial where it is desirable to match children's responses at baseline and follow-up, and as such names are required. All eligible children's names were provided by schools prior to data collections, and entered into a secure password-protected database alongside their individual participant number. This number appeared at the top of all questionnaires distributed to children, and in all data files where the child's responses were entered. At no point was any child's data ever entered alongside their name, and the database of names and participant numbers was used to ensure that the child was given the same identification number at baseline and follow-up.

Thirdly, in order to maximise participation and engagement, methods had to be as child-friendly as possible. As recommended by Petersen et al. [49], data were collected by research staff within the school premises, both in separate rooms with a small number of individuals, and in large group settings. Teachers' presence was requested during the group data collection period, although they were asked not to involve themselves in the data collection procedures. Data collection procedures were thoroughly explained by the researchers, and where possible, used attractively illustrated child-friendly measures, developed for the study. Assurances of anonymity were provided and children were encouraged to ask questions if there was anything that they didn't understand.

Results of this study will be disseminated in a report to the Welsh Assembly Government once all data has been coded, entered, accuracy checked and analysed, and will also appear in a number of academic journal publications. In the meantime, it is hoped that the description offered in this paper is of use to those concerned with the development of further evaluations of similar school-based initiatives.

## Competing interests

The author(s) declare that they have no competing interests.

## Authors' contributions

Principal responsibility for study design was assumed by LM, SM and KT. KT and SM project managed. GM was primarily responsible for day-to-day running of the quantitative side of the evaluation and RL was primarily responsible for day-to-day running of the qualitative process evaluation. GM drafted the manuscript, integrating substantial written contributions from KT. LM conducted sample size calculations. LM and CD designed the analysis plan. JH and CR were responsible for developing the initial research specification and for input into the study design. All authors read and commented on drafts and approved the final manuscript.

## Pre-publication history

The pre-publication history for this paper can be accessed here:


